# Development of an Enzyme-Linked Immunosorbent Assay Based on CD150/SLAM for the Detection of Peste des Petits Ruminant Virus

**DOI:** 10.3389/fvets.2020.00196

**Published:** 2020-04-28

**Authors:** Meera Prajapati, Yongxi Dou, Xueliang Zhu, Shuaiyang Zhao, Niyokwishimira Alfred, Yanmin Li, Zhidong Zhang

**Affiliations:** ^1^State Key Laboratory of Veterinary Etiological Biology, Lanzhou Veterinary Research Institute, Chinese Academy of Agricultural Sciences, Lanzhou, China; ^2^CAAS-ILRI Joint Laboratory for Ruminant Disease Control, Lanzhou Veterinary Research Institute, Chinese Academy of Agriculture Sciences, Lanzhou, China; ^3^Animal Health Research Division, Nepal Agricultural Research Council, Lalitpur, Nepal

**Keywords:** ELISA, PPR virus, SLAM (CD150), diagnosis, RT-qPCR

## Abstract

Peste des petits ruminant (PPR) is an economically important severe viral disease of small ruminants that affects primarily the respiratory and digestive tract. Specific detection of the PPR virus (PPRV) antigen plays an important role in the disease control and eradication program. In this study, an indirect enzyme-linked immunosorbent assay (ELISA) based on the recombinant goat signaling lymphocyte activation molecule (SLAM) as the capture ligand was successfully developed for the detection of the PPRV antigen (PPRV SLAM-iELISA). The assay was highly specific for PPRV with no cross-reactions among foot and mouth disease virus, Orf virus, sheep pox virus, and goat pox virus and had a sensitivity with a detection limit of 1.56 × 10^1^ TCID_50_/reaction (50 μl). Assessment of 136 samples showed that the developed PPRV SLAM-iELISA was well correlated with real-time RT-qPCR assays and commercially available sandwich ELISA for detection of PPRV and showed relative sensitivity and specificity of 93.75 and 100.83%, respectively. These results suggest that the developed PPRV SLAM-iELISA is suitable for specific detection of the PPRV antigen. This study demonstrated for the first time that the goat SLAM, the cellular receptor for PPRV, can be used for the development of a diagnostic method for the detection of PPRV.

## Introduction

Peste des petits ruminant (PPR) is an economically important transboundary animal disease of sheep and goat characterized by fever, stomatitis, conjunctivitis, gastroenteritis, and pneumonia (1). The causative agent PPR virus (PPRV) is a paramyxovirus which is a linear, single-stranded negative-sense RNA virus. Structurally and genetically, this virus is closely related to the rinderpest virus (RPV) of cattle and buffaloes, measles virus (MeV) of humans, distemper virus of dogs and some wild carnivores, and morbilliviruses of aquatic mammals (2). The primary host of this virus is goats and sheep; however, goats seem to be more susceptible than sheep (3). Interestingly, cattle, buffaloes, camels, and pigs have also been reported to develop subclinical infection but do not play any role in excreting the virus (4, 5). Furthermore, the spillover of PPRV to other unusual hosts such as single-humped camels, gazelles, ibex, gemsbok, deer, bushbuck, wild goats, and pigs has become a challenge for disease eradication (6). The morbidity and mortality rates of the disease may reach up to 100 and 90%, respectively (7).

PPRV was first reported in Ivory Coast (Cote d'Ivoire) of West Africa in 1942 (8). Now, the disease has spread in Central and East Africa, the Middle East, Turkey, China, India, and Nepal, reaching Europe's doorstep with cases reported in Morocco (9), Turkey (10), and Georgia (11). If left uncontrolled, it will spread even further, causing massive loss and hardship for millions of farmers and herders. The annual economic losses have been estimated to be up to USD 2.1 billion (12). Considering the need for the control and eradication of the disease, the OIE and FAO have set a joint global eradication strategy by the year 2030. In order to achieve this goal, effective diagnostic tools play a vital role for the timely diagnosis of the disease. Antigen detection methods such as the agar gel immunodiffusion test (AGID)/AGPT, counterimmunoelectrophoresis (CIE), dot enzyme immunoassays, and differential immunohistochemical staining on tissue sections were initial diagnostic methods, but these techniques are less sensitive and unreliable for use in the routine diagnosis of PPR (13). With the advancement of molecular biology, advanced techniques like virus isolation, RT-PCR and its variants, PCR ELISA, and loop-mediated isothermal amplification (LAMP) have been developed (14–17). However, these molecular methods require specialized equipment and skilled personnel for their operation. To overcome these problems, the rapid and sensitive ELISA could be an effective tool for the detection of the PPRV antigen.

The signaling lymphocyte activation molecule (SLAM) also known as CD150, which is a type I membrane protein classified under the CD2 subset of the immunoglobulin superfamily of surface receptors, is the major cellular receptor required for PPRV to attach to the cells, which mediates infection of immune cells and dissemination of the virus (18, 19). Based on this information, SLAM could be exploited as an antigen capture ligand to develop an improved immunoassay for the detection of PPRV. It was reported that the extracellular domain of human SLAM is sufficient to bind with MeV (20), and it is suggested that the extracellular domain of sheep/goat SLAM may be sufficient to interact with PPRV (21). Therefore, in this study, the extracellular domain of goat SLAM was expressed, and then a recombinant goat SLAM (rgSLAM)-based indirect ELISA (PPRV SLAM-iELISA) was successfully developed for detection of the PPRV antigen.

## Materials and Methods

### Cells and Viruses

African green monkey kidney cells (Vero) provided by the Lanzhou Veterinary Research Institute (LVRI), Lanzhou, China, were grown and maintained as monolayers in Dulbecco's Modified Eagles medium (DMEM, Gibco) supplemented with 10% fetal bovine serum (FBS) and 1% penicillin/streptomycin (PS) at 37°C with 5% CO_2_. A PPRV/Nigeria 75/1 strain provided by the LVRI was propagated in monolayer Vero cells maintained in 2% FBS DMEM media. When Vero cells showed >80% infection, the cells were harvested by thawing and freezing for three times and then centrifuged at 400 g for 15 min at 4°C to remove cellular debris (22). The harvested supernatant was filtered through 0.33 μm filters using Amicon filter tubes (Amicon Ultra-15 Centrifugal Filters, Lot: R8KA00411). The purified PPRV preparation was aliquoted and stored at −80°C until use. All the other viruses used in this study were provided by LVRI: Orf virus (ORFV)/Vaccine/CHA; goat pox virus (GPV) AV40, sheep pox virus (SPV) Gulang2009; foot-and-mouth disease virus (FMDV)/O/CHA; FMDV/A/CHA.

### Construction of Cloning and Expression Vector

The PET SUMO vector was obtained from Wuhan Gene Create Biological Engineering Co., Ltd, China. The goat SLAM gene sequence was obtained from GenBank (access no. NM_001285726.1). The signal and transmembrane peptide of the amino acid sequence was analyzed by Signal IP-4.1 and TMHMM 2.0 online software. The extracellular region of the goat SLAM amino acid sequence (29–237 aa) and goat SLAM coding nucleotide sequence is shown in Table 1.

**Table 1:** Amino acid and nucleotide sequences of SLAM and SUMO.

**Amino acid sequences**	
His-SUMO	HHHHHH-MSDSEVNQEAKPEVKPEVKPETHINLKVSDGSSEIFFKIKKTTPLRRLMEAFAKRQGKEMDSLRFLYDGIRIQADQTPEDLDMEDNDIIEAHREQIGG
SLAM	LTSSTKTIRGQLGSSVLLPLASEEISRSMNKSIHILVTMAESPRDTVKKKIVSLDLRKGDSPRLEDGYEFHLENLSLRILKSRKEDEGWYFISLEENVSVQHFSLQLKLYEQVSTPQIKVLNSTQEDGNCSLMLACVVEKGDHVTYNWSEEAGAPLLSPTNSSHLLYLTLGPQHANNVYICIASNPISNSSQTFIPWSRCSSRPPESRQ
**Nucleotide sequences**	
His-SUMO	ATGGCTCACCATCATCATCATCAT-ATGTCGGACTCAGAAGTCAATCAAGAAGCTAAGCCAGAGGTCAAGCCAGAAGTCAAGCCTGAGACTCACATCAATTTAAAGGTGTCCGATGGATCTTCAGAGATCTTCTTCAAGATCAAAAAGACCACTCCTTTAAGAAGGCTGATGGAAGCGTTCGCTAAAAGACAGGGTAAGGAAATGGACTCCTTAAGATTCTTGTACGACGGTATTAGAATTCAAGCTGATCAGACCCCTGAAGATTTGGACATGGAGGATAACGATATTATTGAGGCTCACAGAGAACAGATTGGTGGT
SLAM	CTGACCAGCAGCACCAAAACCATTCGTGGTCAGCTGGGTAGCAGCGTTCTGCTGCCGCTGGCAAGCGAAGAAATTAGCCGTAGCATGAATAAAAGCATCCATATTCTGGTTACCATGGCAGAAAGTCCGCGTGATACCGTTAAAAAGAAAATTGTTAGCCTGGATCTGCGCAAAGGTGATAGTCCGCGTCTGGAAGATGGTTATGAATTTCATCTGGAAAATCTGAGCCTGCGCATTCTGAAAAGCCGTAAAGAAGATGAAGGCTGGTATTTCATTTCCCTGGAAGAAAATGTGTCCGTGCAGCATTTTAGCCTGCAGCTGAAACTGTATGAACAGGTTAGCACACCGCAGATTAAAGTTCTGAATAGCACCCAAGAAGATGGTAATTGTAGCCTGATGCTGGCATGTGTTGTTGAAAAAGGTGATCACGTTACCTATAATTGGAGCGAAGAAGCAGGCGCACCGCTGCTGAGCCCGACCAATAGCAGCCATCTGCTGTATCTGACCCTGGGTCCGCAGCATGCAAATAATGTGTATATTTGTATTGCGAGCAACCCGATTAGCAATAGCAGTCAGACCTTTATTCCGTGGTCACGTTGTAGCAGCCGTCCGCCTGAAAGCCGTCAGTAA

The segment of the goat SLAM gene which encodes the extracellular domain was synthesized and successfully cloned into PET SUMO vector via *Bam*HI and *Xho*I restriction enzyme infusion with the PET SUMO His-tag. The His-tag was at the N terminal with normal hexa-histidine (HHHHHH), followed by SUMO and then SLAM. The total size of the phagemid was 6,209 bp, and the molecular weight of the recombinant protein is 42 kDa. The direction of the clone was identified by double digestion and DNA sequencing. The recombinant plasmid was then used to transform in *Escherichia coli* competent cells (Rosetta), and the transformed cells were cultured at 37°C in Luria–Bertani (LB) medium plate containing 50 μg/ml of kanamycin. The single colony of freshly transformed *E. coli* containing the constructed plasmid was cultured in 3 ml of LB liquid medium containing 50 μl/ml of kanamycin and incubated at 37°C until the optical density (OD) at 600 nm reached 0.6. Then the expression of the fusion protein was induced by isopropyl-β-d-thiogalactopyranoside (IPTG) and analyzed by SDS-PAGE. The fusion protein expression was induced massively and purified by Ni-affinity chromatography.

### SDS-PAGE and Western Blot Analysis

The molecular weight of the recombinant protein was analyzed by SDS-PAGE and western blot according to the standard protocol (22). Briefly, the recombinant protein was subjected to SDS-PAGE with 12% resolving gel and 5% stacking gel. The protein was then transferred to a polyvinylidene difluoride (PVDF) membrane (Immobilon-PSQ membrane) and blocked in blocking buffer for 2–3 h at room temperature. The membrane was washed five times in Tris-buffered saline with Tween-20 (TBST) buffer. Next, the mouse anti-His monoclonal antibody (Huamei Company, China) was added in 1:1,000 dilution and incubated for 1 h at room temperature. Subsequently, the membrane was washed and incubated with horseradish peroxidase (HRP)-labeled sheep anti-mouse in 1:10,000 for 1–2 h at room temperature. Then the membrane was washed and color was developed using an Immobilon western chemiluminescent HRP substrate (Immobilon, USA).

### Preparation of PPRV Antisera

The positive serum used as the primary polyclonal antibody for this study was obtained from sheep immunized with the PPRV Nigeria 75/1 vaccine strain. Sheep were kept at the experimental unit of LVRI, Lanzhou, Gansu, China, in accordance with the instructions and guidelines of the animal ethics committee (permit no. LVRIAEC-2018-001), which were approved by the People's Republic of China. Sheep were immunized three times at 2-week intervals (23). Sera were collected and checked for PPRV antibody by PPRV c-ELISA for N antibody detection (ID Vet, France). The positive sera were optimized for the development of PPRV SLAM-iELISA.

### Optimization of Coating Buffer, Blocking Buffer, and rgSLAM

To optimize the optimum conditions of the PPRV SLAM-iELISA, the purified PPRV preparation was used as a positive control and the TBScm buffer as negative controls. Different concentrations of the rgSLAM, coating buffer, and blocking buffer were selected and optimized. For the selection of the appropriate coating buffer, two types of coating buffer, (i) TBScm with pH 7.6 and (ii) sodium bicarbonate/carbonate salts with pH 9.6, were used for the dilution of the rgSLAM and coated overnight at 4°C. The next day, ELISA was performed, and the P/N value was calculated to interpret the results.

In order to reduce the background interference and improve the signal-to-noise ratio, different blocking buffers such as 5% skimmed milk powder, 1% casein in TBScm, and 1% casein in TBScm + 2% normal bovine serum (NBS) were applied and selected based on the P/N value. Similarly, the rgSLAM was diluted in 2-fold dilution, and its working concentration was optimized.

### Indirect ELISA

A purified rgSLAM protein was used to coat microtiter ELISA wells at pre-optimized concentrations followed by overnight incubation at 4°C. The next day, the wells were washed with PBS containing 0.05% Tween-20 (PBST) for four times with gentle shaking. The plates were blocked with a pre-optimized blocking buffer, that is, 1% casein in TBScm (0.85% saline with 0.02 M Tris, 0.002 M CaCl_2_, and 0.001 M MgCl_2_, pH value 7.6) buffer for 1 h at 37°C, and washed four times by PBST. PPRV-positive and PPRV-negative control samples were diluted in TBScm buffer at a proper concentration and were used for checkerboard titration. The plates were incubated at 37°C for 1 h and washed four times as before. Thereafter, 50 μl of PPRV antisera at a pre-optimized dilution was added and incubated for 1 h at 37°C. Next, the antigen/antibody complex was detected by using rabbit polyclonal antibody against goat IgG. Finally, the substrate was added followed by addition of a stop solution. The absorbance values were read at 450 nm in an ELISA reader machine (SpectraMax Plus, USA). The results were expressed as the ratio of the OD values between the test samples and the negative control (S/N). For the positive control, the ratio was expressed as P/N.

### Samples

A total of 120 lymph nodes from slaughterhouses of Lanzhou, Gansu province, were collected and used for the detection of PPRV. Likewise, a total of 14 tissue samples (lymph nodes, heart, liver, spleen, and kidney) from PPR-suspected sheep in Gansu province during postmortem were collected, packed in plastic ziplock bags, and then transported to the laboratory on ice. The samples were stored at −70°C until the time of processing. Tissue samples were cut into a small pea size and rinsed with PBS (7.6) to remove any blood particles. Tissues were further cut into small pieces with the help of sterile scissors, and PBS was added to the fine samples to make 10% tissue suspension and incubated overnight at 4°C. Samples were tested by the developed PPRV SLAM-iELISA for the detection of PPRV.

### Real-Time RT-qPCR Assay

Viral RNA was extracted using a viral RNA/DNA extraction kit (TaKaRa MiniBEST, viral RNA/DNA extraction kit Ver. 5.0 Lot: AIF2421A). Real-time RT-qPCR assay was performed in an Agilent Technologies Stratagene Mx3005P thermocycler (Life Technologies, USA) using a SuperScript™ III Platinum One-Step qRT-PCR kit (Invitrogen, Lot: 2006295). A TaqMan probe and primers designed for the N protein of PPRV were used as previously described (24, 25). The program was run at 50°C for 15 min, followed at 95°C for 10 min, 95°C for 15 s, and 60°C for 1 min at 40 cycles. The data were analyzed using the Mx3005P System software.

## Results

### Expression and Purification of rgSLAM Protein

**Figure 1:**
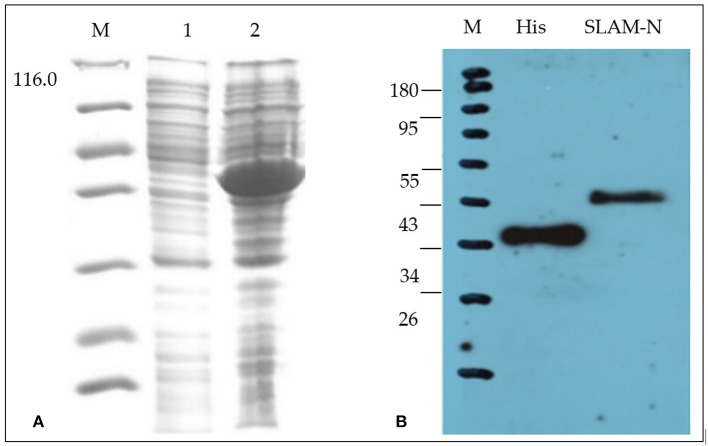
Characterization of recombinant goat SLAM protein (rgSLAM). **(A)** Expression of the recombinant SLAM-PETSUMO using Rosetta strains. M: ladder; lane 1: uninduced bacteria; lane 2: induced SLAM-PET SUMO. **(B)** Analysis of the recombinant SLAM-PET SUMO by western blot.

Results of SDS-PAGE and western blot analysis (Figure 1) showed that the recombinant protein expressed by the *E. coli* expression system has the correct molecular weight, and the total amount of the recombinant protein after purification is 5 mg/500 ml with a purity of 90%.

### Optimization of Coating Buffer, Blocking Buffer, and rgSLAM

**Figure 2:**
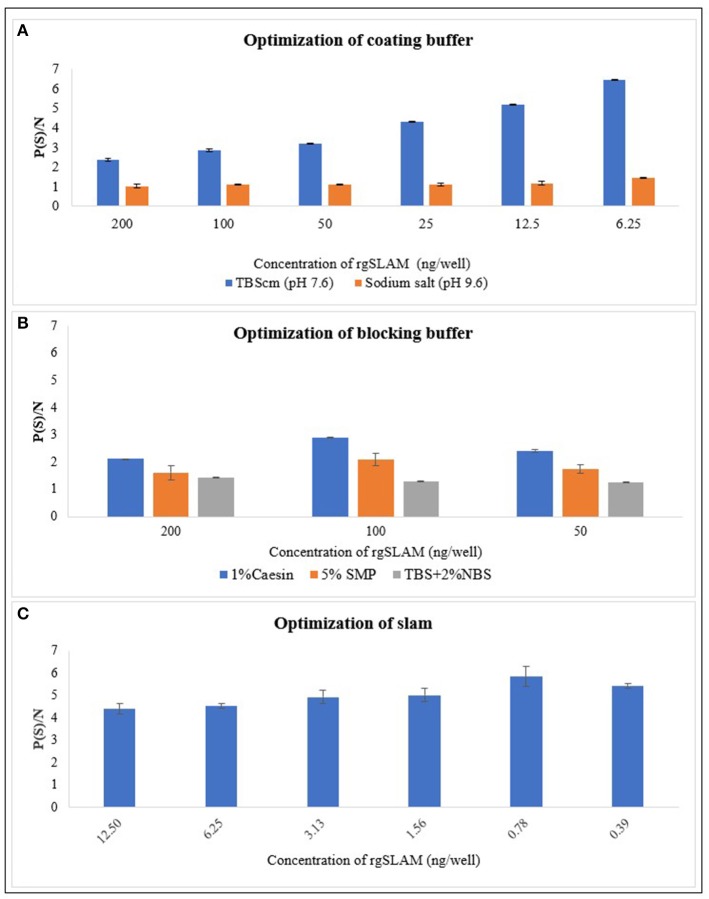
Optimization of the parameters of rgSLAM-based indirect ELISA. **(A)** Optimization of coating buffer. Different concentrations of the rgSLAM were coated in TBScm coating buffer having a pH value of 7.6 and in sodium carbonate/bicarbonate salts having a pH value of 9.6, and the P/N values were compared. The blue line represents the P/N value obtained with the TBScm buffer, and the orange line represents the sodium salt coating buffer. **(B)** Optimization of the blocking buffer; 1% casein, Tris-based solution with 2% normal bovine serum (TBS + 2% NBS), and 5% skimmed milk powder (SMP) were used. **(C)** Optimization of the rgSLAM using different concentrations. The highest dilution of SLAM that exhibited maximum difference between positive and negative samples (P/N) was used further for testing field samples. rgSLAM, recombinant goat SLAM; TBScm, Tris-based solution with calcium and magnesium.

The results of the optimized coating buffer are shown in Figure 2A. As the TBScm buffer exhibited the maximum P/N value compared with the sodium salt buffer, the TBScm coating buffer having a pH value of 7.6 was chosen as the coating buffer for the rgSLAM. Likewise, the results of comparison of different blocking buffers showed that 1% casein in the TBScm buffer produces the least non-specific binding for antigen detection ELISA (Figure 2B). The result of the optimum working concentration of rgSLAM (Figure 2C) showed that the highest dilution at which the maximum difference with respect to OD between positive and negative controls produced was at 0.39 ng/well. Hence, the different parameters of the PPRV SLAM-iELISA such as rgSLAM were optimized at the concentration of 0.4 ng/well, with the HRP-conjugated rabbit polyclonal antibody to goat IgG dilution at 1:40,000 and 1% casein in TBScm as the blocking buffer. These optimized conditions were used for the rest of the ELISA experiments.

### Determination of the Threshold (Cut-Off) Value

**Figure 3:**
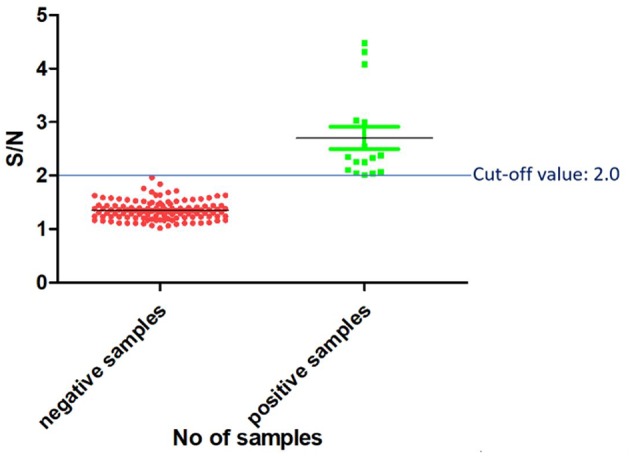
Cut-off value of SLAM-based iELISA.

Once the reagents used in the PPRV SLAM-iELISA were optimized and standardized, samples of known status were used to estimate the cut-off value of the assay. The results were expressed as the ratio of the mean OD value of the samples and the mean OD value of the negative control (S/N). Altogether 120 known-negative samples were confirmed as negative for PPRV by RT-qPCR, and 16 known-positive samples were confirmed as positive by RT-qPCR. These samples were then used for the determination of the cut-off value. As shown in Figure 3, the cut-off value was set at an S/N ratio of 2. All the positive samples were tested positive, and negative samples were tested negative when this cut-off value was chosen.

### Analysis of the Sensitivity and Specificity of PPRV SLAM-iELISA

To test the sensitivity of PPRV SLAM-iELISA, serial dilutions of PPRV (ranging from 0.5 × 10^3^ to 1.9 TCID_50_/50 μl) were tested in replicates. As shown in Table 2, the dynamic detection range of the assay spans from 0.5 × 10^3^ TCID_50_/50 μl to 1.5625 × 10^1^ TCID_50_/50 μl with a detection limit of 1.5625 × 10^1^ TCID_50_/50 μl of PPRV.

**Table 2:** Analytical sensitivity of the PPRV SLAM-iELISA.

**PPRV dilution**	**TCID_**50**_/50 μl**	**S/N ± SD**	**Results**
Undiluted	0.5 × 10^3^	8.967 ± 0.126	P
1:8	0.06251 × 10^3^	5.699 ± 0.111	P
1:16	3.125 × 10^1^	3.673 ± 0.029	P
1:32	1.5625 × 10^1^	2.011 ± 0.06	P
1:64	7.8 × 10^0^	1.394 ± 0.016	N
1:128	3.91 × 10^0^	1.001 ± 0.002	N
1:256	1.9 × 10^0^	0.973 ± 0.0006	N

Evaluation of the specificity of the PPRV SLAM-iELISA was done by cross detection of other viruses which infect the epithelium or mucus in sheep and goats, including FMDV O, FMDV A, ORV, SPV, and GPV. No cross reaction was observed, indicating the assay was specific for PPRV (Table 3).

**Table 3:** Cross-reactivity assessment of SLAM-based ELISA.

**SN**	**Samples**	**S/N ± SD**	**Result**
1	PPRV	5.638 ± 0.023	P
2	Orf	1.620 ± 0.014	N
3	FMD “O”	1.041 ± 0.026	N
4	FMD “A”	0.982 ± 0.0007	N
5	SPV	0.710 ± 0.007	N
6	GPV	0.644 ± 0.011	N

### Performance of PPRV SLAM-iELISA on Clinical Samples

A total of 136 samples which include 120 known-negative samples and 16 known-positive samples were tested simultaneously by PPRV SLAM-iELISA, commercially available sandwich ELISA, and RT-qPCR (Table 4). Fifteen samples were determined to be positive by PPRV SLAM-iELISA while 16 samples were positive by both sandwich ELISA and RT-qPCR (CT value ranging from 19.6 to 32.9). The relative sensitivity and specificity of PPRV SLAM-iELISA for detection of the PPRV antigen were 93.75 and 100.83%, respectively, when compared with commercially available sandwich ELISA and RT-qPCR.

**Table 4:** Performance of PPRV SLAM-iELISA on clinical samples in comparison with PPRV RT-qPCR and Sandwich ELISA (ID VET).

**Sample type**	**No. of samples tested**	**PPRV SLAM-iELISA**		**RT-qPCR**		**Sandwich ELISA**	
		**Positive**	**Negative**	**Positive**	**Negative**	**Positive**	**Negative**
Lymph nodes	124	4	120	4	120	4	120
Lungs	3	3	0	3	0	3	0
Liver	2	2	0	2	0	2	0
Kidney	2	2	0	2	0	2	0
Spleen	3	3	0	3	0	3	0
Heart	2	1	1	2	0	2	0
Total	136	15	121	16	120	16	120

## Discussion

PPR is an acute infectious disease of sheep and goats which is spreading at an alarming rate across the world. The prevention, control, and eradication of the disease depend upon appropriate diagnostic methods and timely vaccination of susceptible animals. ELISA is one of the robust immunological diagnostic methods for detecting antibodies or antigens. ELISA tests developed for PPR diagnosis are specially designed for detection of antibody against the N or H protein with variable sensitivity and specificity. However, this study used the rgSLAM, which is a major receptor for PPRV, as a capture ligand for the development of an indirect ELISA for detecting PPRV and demonstrated for the first time that the PPR viral receptor protein can be used for the diagnostic test. This study shows that the developed PPRV SLAM-iELISA is specific for the detection of PPRV. The extracellular V domain of SLAM is mainly responsible for the PPRV binding site, so the extracellular domain SLAM encoding gene was selected for expression and used as a coating capture ligand to detect PPRV. The ELISA parameters were optimized to get the maximum difference between positive control and negative control. This assay has 93.75% sensitivity and 100.83% specificity in comparison with RT-qPCR and ID Vet sandwich ELISA. Moreover, repeated thawing and freezing of the rgSLAM should be avoided as much as possible to get good sensitivity. Even though only few positive tissue samples, that is, 16 in number, were available for the test, it proves that the rgSLAM expressed in this study can be used successfully for the development of an ELISA for the detection of PPRV. Similarly, the analytical specificity of the test showed that it did not cross react with related diseases of sheep and goats. The lower sensitivity of the developed PPRV SLAM-iELISA can be well-justified by the following maxim: the “diagnostic sensitivity of a test is inversely proportional to reduction in the specificity” (26).

Even though this PPRV SLAM-iELISA is less sensitive than molecular techniques such as RT-qPCR, the benefit of this ELISA is its capability to detect the whole virus particle rather than a fragment of the gene. It also does not require extensive procedures and equipment like in RT-qPCR. Above all, although RT-PCR and RT-qPCR are very sensitive, they sometimes give false-positive results and requires that the quality of sample be free from contamination. In that case, this ELISA could be an ideal alternative to RT-qPCR for PPRV detection. In addition, ELISA is quicker, easier, and simpler to perform than PCR. The developed PPRV SLAM-iELISA will be more useful, especially in developing countries which cannot afford the expense of RT-qPCR and cell culture facilities. Hence, this SLAM-iELISA is highly sensitive and specific, is easier to produce and perform, is suitable for screening large sample sizes, and has better documentation of evidence-based clinical sample status in PPR endemic settings.

Although virus isolation is a gold standard test for PPR virus detection, it is time-consuming, which requires cell culture facilities that are relatively complex. Other antigen detection methods such as the immunochromatographic test developed for rapid detection of H protein by Baron et al. (27) are rapid, but the sensitivity and specificity of the test in relation to RT-PCR are 85% and 95% (27). Our ELISA results agreed very well with RT-qPCR and commercial sandwich ELISA and have proven to be suitable for testing tissue samples for antigen detection. Unfortunately, due to lack of nasal and fecal swab samples from infected animals, this assay could not test nasal swab and fecal samples of infected animals. The developed PPRV SLAM-iELISA can also be safely used in endemic and non-endemic countries and specially during massive outbreak of the disease where all the samples can be tested at a time without any difficulty. This study is likely to contribute to the serological diagnosis of PPR in resource-limited settings to smooth the progress of early implementation of control measures including quarantine, vaccination, and possibly stamping out.

## Conclusion

This study successfully developed an rgSLAM-based indirect ELISA for the detection of PPRV. This assay should further be fully evaluated using different types of clinical samples.

## Data Availability Statement

The datasets used and/or analyzed during the current study are available from the corresponding author on reasonable request.

## Ethics Statement

The animal study was reviewed and approved by Lanzhou Veterinary Research Institute (LVRI), Chinese Academy of Agricultural Sciences (CAAS), Lanzhou, Gansu, P.R. China in accordance with the instructions and guidelines of the animal's ethics committee (permit no. LVRIAEC-2018-001).

## Author Contributions

ZZ, YL, and MP designed the study. MP performed the experiment. YL and ZZ supervised the experiment and revised the manuscript. YD, XZ, SZ, and NA contributed the materials. MP drafted the manuscript and was the major contributor in writing the manuscript. All authors read and approved the manuscript.

## Conflict of Interest

The authors declare that the research was conducted in the absence of any commercial or financial relationships that could be construed as a potential conflict of interest.
